# Analyzing Spatial Dependency of the 2016–2017 Korean HPAI Outbreak to Determine the Effective Culling Radius

**DOI:** 10.3390/ijerph18189643

**Published:** 2021-09-13

**Authors:** Kwideok Han, Meilan An, Inbae Ji

**Affiliations:** 1 Department of Institutional Research and Analytics, Oklahoma State University, Stillwater, OK 74078, USA; kwideok.han@okstate.edu; 2 Department of Food Industrial Management, Dongguk University, Jung-gu, Seoul 04620, Korea; zhensee@naver.com

**Keywords:** highly pathogenic avian influenza, HPAI, spatial random effects logistic model, spatial dependency, spatial autocorrelation, effective culling radius

## Abstract

Highly pathogenic avian influenza (HPAI) outbreaks are a threat to human health and cause extremely large financial losses to the poultry industry due to containment measures. Determining the most effective control measures, especially the culling radius, to minimize economic impacts yet contain the spread of HPAI is of great importance. This study examines the factors influencing the probability of a farm being infected with HPAI during the 2016–2017 HPAI outbreak in Korea. Using a spatial random effects logistic model, only a few factors commonly associated with a higher risk of HPAI infection were significant. Interestingly, most density-related factors, poultry and farm, were not significantly associated with a higher risk of HPAI infection. The effective culling radius was determined to be two ranges: 0.5–2.2 km and 2.7–3.0 km. This suggests that the spatial heterogeneity, due to local characteristics and/or the characteristics of the HPAI virus(es) involved, should be considered to determine the most effective culling radius in each region. These findings will help strengthen biosecurity control measures at the farm level and enable authorities to quickly respond to HPAI outbreaks with effective countermeasures to suppress the spread of HPAI.

## 1. Introduction

Highly pathogenic avian influenza (HPAI) is an acute contagious disease with an exceptionally high propagation rate that affects poultry as well as, potentially, humans. Accordingly, the World Organization for Animal Health (Office International des Epizooties, OIE) classifies HPAI as a disease subject to management. Outbreaks of HPAI have occurred in several countries worldwide and the global spread of this disease has been mainly attributed to migratory birds [1,2]. The initiation of HPAI outbreaks in Korea is also associated with the arrival of migratory birds [3].

Once an outbreak occurs, factors contributing to HPAI spread among farms include poultry breeding scale; proximity of farms to roads, lakes, wetlands, and slaughterhouses; proximity of farms to habitats of migratory birds; and farm elevation [4,5,6,7,8,9,10,11,12,13,14]. The mechanism of the spread of HPAI can be explained via direct or indirect contact with infected materials, vehicles, or persons through a contact network [15]. Research also suggests that the spread of HPAI between farms may occur by airborne transmission [16]. Accordingly, the risk of HPAI infection is greater in farms located in proximity to an infected farm [17]. As such, infectious diseases such as HPAI have the characteristic of forming clusters in space [7,8,9,18,19,20].

Since the first occurrence of HPAI in Eumseong, Chungbuk, Korea, in 2003, HPAI outbreaks have occurred intermittently every two to three years throughout the 2000s and every year in the 2010s. Korea has categorized HPAI as a “Type 1 infectious disease” and the implementation of quarantine measures to control the spread of HPAI is critical to minimize the damage caused by HPAI. Once HPAI is detected, the area surrounding the infected farm is subject to Emergency Quarantine Measures (Implement quarantine measures against Avian Influenza (AI)) at https://www.mafra.go.kr/english/1441/subview.do and were accessed on 10 January 2020. An Emergency Quarantine Measure divides the infected region into three zones: an infected zone, a buffer zone, and a surveillance zone. The infected zone is within a 500 m radius of the HPAI infected farm. The buffer zone refers to where HPAI may spread and is within a 500 m to 3 km radius from the HPAI infected farm. The surveillance zone, within a 3–10 km radius of an HPAI infected farm, is where monitoring and measures to prevent further spread of HPAI are promoted.

Different measures within the zones are implemented to contain the spread of HPAI including culling livestock from the infected area. Not only is livestock culled from the original farm infected by HPAI, but also from surrounding farms up to a 3 km radius. However, the culling radius has not been consistent, and different culling radii have been used as control measures [21]. Before 2017, the culling radius was 500 m. In the seventh HPAI outbreak of 2016–2017, the radius was extended to 3 km and led to the culling of 37.8 million poultry from 946 poultry farms. More recently, during the 2020–2021 HPAI outbreak, the culling radius was adjusted from 3 km to 1 km and then eliminated for farms strictly adhering to quarantine protocols.

Consequently, the livestock industry, along with related industries, have been significantly impacted. During the 2016–2017 HPAI outbreak, the South Korean Government incurred losses of KRW 300.7 billion (about USD 273 billion), the highest fiscal loss in Korean history related to HPAI outbreaks to date [22]. These fiscal losses only refer to direct losses, such as compensation for culling and the quarantine costs of farm households. If additional losses in related industries, such as feed, meat processing, and food service are included, the economic damage caused by HPAI outbreaks is considerably greater. Due to such large-scale economic damages and the different culling radii implemented, both poultry farms as well as the government have raised concerns about providing a more scientific rationale for the most effective culling range. However, little research has investigated the proper culling range to contain HPAI outbreaks in Korea until recently [21,23].

This study analyzes the risk factors associated with the probability of a farm being infected with HPAI during the 2016–2017 HPAI outbreak. To account for spatial dependence, a spatial econometrics technique was used. The main objective of this study is to determine the effective culling radius based upon the predicted probability of a farm being infected by neighboring HPAI-infected farms. This would help policymakers implement emergency control measures that would most effectively contain the HPAI outbreak while minimizing economic losses.

## 2. Materials and Methods

### 2.1. Data

Information about individual poultry farms during the seventh HPAI outbreak (from 16 November 2016 to 19 June 2017) was obtained as of January 2018 from the Korea Animal Health Integrated System (KAHIS) administered by the Ministry for Food, Agriculture, Forestry, and Fisheries at https://home.kahis.go.kr/home/lkntscrinfo/selectLkntsOccrrncList.do and was accessed on 10 January 2020. Data on the geographical characteristics of the farms were collected via the Arc-GIS program.

After excluding farms located on islands such as Jeju Island and Ulleung-do and farms raising other poultry (quails and geese), a total of 6890 poultry farms were examined. Of the 6890 farms, 401 farms were reported as being infected with HPAI, accounting for 5.8% of the total number of poultry farms analyzed (Ministry of Agriculture, Food and Rural Affairs; https://www.mafra.go.kr/FMD-AI2/2179/subview.do, last accessed on 10 January 2020). Considering the type of livestock, proportionally breeder duck farms had the highest incidence of HPAI infection (25.0%) followed by laying hen farms (13.3%), broiler duck farms (4.0%), and broiler hen farms (3.8%). Breeder hen farms were not infected during the 2016–2017 HPAI outbreak. The number of monitored farms was 168 which is 2.4% of all farms included (Table 1).

The total number of poultry raised by individual farms (HEADS), the total number of same poultry breed heads in a county (BREED HEADS), and the total number of poultry reared in a county (COUNTY HEADS) were collected. BREED HEADS and COUNTY HEADS are the total numbers from the county that the individual farm belongs. Using the Arc-GIS program, data on the number of farms or the number of poultry heads in the infected zone (within the 500 m radius), out to the buffer zone (within the 3 km radius), and out to the surveillance zone (within the 10 km radius) were collected. Additionally, we indicated whether a farm was located within a “mass breeding site” (MONITORED) using a dummy variable (1 = located in and 0 = not located in a mass breeding site). “Mass breeding sites” are defined as ≥10 farms within a 500 m radius or ≥20 farms within a 1 km radius and are monitored regularly for the presence of HPAI. A total of 14 areas were designated as “mass breeding sites” (Implement quarantine measures against Avian Influenza (AI) (mafra.go.kr) (accessed on 10 January 2020) in the dataset used. Of the total 401 HPAI infected farms, 40 farms were located in “mass breeding sites”, accounting for 10% of the total number of HPAI-infected farms.

Using the Arc-GIS program, the distance of each farm to the closest two-lane road, rivers and lakes, feed markets, slaughterhouses, and habitats of migratory birds, as well as altitude above sea level, were collected. The descriptive statistics of the variables are reported in Table 2.

### 2.2. Logistic Regression Model

A logistic regression model was used to estimate the probability of, and determine the factors influencing, a farm being infected with HPAI. The dependent variable was classified into one of two groups: “Yes” (a farm infected with HPAI) and “No” (a farm not infected with HPAI). The logistic regression model is written as:

(1) $$\mathrm{logit}\left(y_{i}\right)=\log\left(\frac{P\left(y_{i}=1\right)}{1-P\left(y_{i}=1\right)}\right)=\overset{K}{\underset{k=1}{\sum }}\beta_{k}x_{k},$$

where $P\left(y_{i}=1\right)$ is the probability that farm *i* is infected with HPAI, $x_{k}$ denotes a vector of the characteristics for each farm, and $\beta_{k}$ are the parameters to be estimated.

To test whether spatial autocorrelation exists in the residuals of the logistic regression model (1), a Moran’s *I* test was conducted as follows [24]:

(2) $${\mathrm{Moran}}^{’}s\;I=\frac{n}{\sum_{i=1}^{n}\sum_{j=1}^{n}w_{ij}}\frac{\sum_{i=1}^{n}\sum_{j=1}^{n}w_{ij}\left(y_{i}-\bar{y}\right)\left(y_{j}-\bar{y}\right)}{\sum_{i}{\left(y_{i}-\bar{y}\right)}^{2}},$$

where *n* is the number of farms, $w_{ij}$ is a spatial weight between farms *i* and *j*, $\sum_{i=1}^{n}\sum_{j=1}^{n}w_{ij}$ is the aggregate of all the spatial weights, $y_{i}$ and $y_{j}$ denote the residual for farm *i* and farm *j*, respectively, and $\left(y_{i}-\bar{y}\right)$ and $\left(y_{j}-\bar{y}\right)$ are the deviations of the residual of farm *i* and farm *j*, respectively, from the mean residual. The spatial weight matrix was created using the 5 nearest neighbors for each farm as described in An et al. [14].

### 2.3. Spatial Random Effects Logistic Model

Based on the Moran’s *I* test, to better estimate the probability of a farm being infected with HPAI incorporating the spatial dependence, a spatial mixed-effects logistic model may be used [25,26,27]. The spatial mixed-effects logistic model is given as:

(3) $$\mathrm{logit}\left(y_{i}\right)=\log\left(\frac{P\left(y_{i}=1\right)}{1-P\left(y_{i}=1\right)}\right)=\overset{K}{\underset{k=1}{\sum }}\beta_{k}x_{k}+g\left(s_{i};\theta \right),$$

where g(·;θ) is a smoothed function of the spatial location of farm *i*, $s_{i}$, which is parameterized by θ. In this study, a spatial random effects logistic model was used to account for the spatially correlated random effects. This model was implemented, assuming a Martérn covariance for the random effects term, g(·) in model (3), as suggested in Kammann and Wand [28] as follows:

(4) $$C\left(r\right)=\frac{1}{\Gamma \left(\nu \right)2^{\nu -1}}{\left(\frac{2\sqrt{\nu r}}{\rho }\right)}^{\nu }{ℵ}_{\nu }\left(\frac{2\sqrt{\nu r}}{\rho }\right),$$

where r is the geographical distance between pairs of farms, ν represents the smoothness parameter, ρ is the scale (correlation decay) parameter, Γ is the gamma function, and ${ℵ}_{\nu }\left(\cdot \right)$ is the modified Bessel function of the second kind, whose order is the differentiability parameter, ν>0 [27].

The predictive accuracy of the spatial random effects logistic model was assessed by comparing the predicted values with the actual data. A farm was classified as “infected” with HPAI if the estimated probability was ≥0.5 and “not infected” with HPAI if the estimated probability < 0.5.

(5) Accuracy=(TP+TN)/(TP+FN+TN+FP),

where *TP* is a true positive, *TN* is a true negative, *FN* is a false negative, and *FP* is a false positive.

### 2.4. Determination of Effective Culling Radius

To determine the most effective culling radius, Moran’s *I* statistic was used by measuring spatial autocorrelation of the estimated probability of a farm being infected with HPAI among neighboring farms with respect to the geographical distance between pairs of farms after controlling for the spatially correlated random effects. The Moran’s *I* statistic given in Equation (2) is modified as follows:

(6) $${\mathrm{Moran}}^{’}s\;I=\frac{n}{\sum_{i=1}^{n}\sum_{j=1}^{n}w_{ij}}\frac{\sum_{i=1}^{n}\sum_{j=1}^{n}w_{ij}\left(p_{i}-\bar{p}\right)\left(p_{j}-\bar{p}\right)}{\sum_{i}{\left(p_{i}-\bar{p}\right)}^{2}},$$

where $p_{i}$ and $p_{j}$ are the predicted probability of farm *i* and farm *j*, respectively, being infected with HPAI, and $\left(p_{i}-\bar{p}\right)$ and $\left(p_{j}-\bar{p}\right)$ denote the deviations of the probability that farm *i* and farm *j*, respectively, is infected with HPAI from the mean probability. In this case, the spatial weight matrix, $w_{ij}$, was constructed using the geographical distance between pairs of farms *i* and *j* as calculated from longitude and latitude coordinates of the center of each farm. The spatial autocorrelation of the probability of a farm being infected with HPAI was examined in 100 m intervals starting from 0.5 km up to 3.2 km. The *R* 3.6.1 program with the package ‘spaMM’ was utilized for all statistical analyses [29].

## 3. Results

### 3.1. Risk Factors for the Likelihood of HPAI Infection

Table 3 presents the results of the logistic regression and spatial random effects logistic models for the probability of a farm being infected with HPAI. The two models provide inconsistent results. The directions and statistical significance of the estimated coefficients for risk factors associated with HPAI infection differ. The logistic regression model shows several factors related to proximity (ROAD, FEED, and SLAUGHTER) and density (HEADS10km, FARMS3km, and FARMS10km) as significant, as has been found in previous studies [12] and references therein.

However, the Moran’s *I* test for spatial dependence of the residuals from the logistic regression model (Moran’s *I* statistic = 0.3054, *p*-value < 0.01) suggests that there is significant spatial correlation among the residuals at the 1% level. If this spatial correlation is not controlled for, then the estimated coefficients are biased, and the significance tests are inconsistent due to heterogeneity of variance [30]. Therefore, the spatial random effects logistic model is more appropriate for this analysis. Once the spatially correlated random effects were accounted for, these factors found to be significant in the logistic regression model, such as proximity to a road, feed market, or slaughterhouse, were no longer significant.

The spatial random effects logistic model suggests that only the risk factors, HEADS, BREED HEADS, HEADS3km, MONITORED, MIGRATORY and ALTITUDE, were significantly associated with the likelihood of a farm being infected with HPAI. In terms of livestock, the total number of poultry at each farm (HEADS) and the total number of the same breed of poultry (BREED HEADS) within a county are positively associated with a higher risk of HPAI infection. In contrast, the total number of poultry reared in a county (COUNTY HEAD), and the farm density (FARM500m, FARM3km, FARM10km) or poultry density (HEAD500m, HEAD10km) within two of the three zones designated by the Emergency Quarantine Measure, are not significantly associated with increased risk of HPAI infection. Only the number of poultry heads within the 3 km buffer zone (HEAD3km) was statistically significant at the 10% level. This contrasts with what has been found in a recent study of the same outbreak [23], and in previous studies [12]. Lee et al. [23] focused on a small region of the overall outbreak, and the results may differ due to the scope of this study being nationwide. In this study, farm density within the zones implemented by the Emergency Quarantine Measure had no effect on the likelihood of HPAI infection of a farm, and poultry density was only a significant factor within the 3 km buffer zone.

The distance of poultry farms from the habitats of migratory birds (MIGRATORY) is negatively associated with a higher risk of HPAI infection at the 5% level indicating that farms closer to migratory bird habitats are more vulnerable to HPAI. Moreover, it was found that farms designated as being in “mass breeding sites” (MONITORED) had a higher probability at the 5% significance level of HPAI infection than farms not designated as being in “mass breeding sites”. This differs from what was found in this study when considering the density of farms, especially that of the number of farms within a 500 m radius (FARMS500m), which were not significantly associated with a greater risk of HPAI infection.

Farms located at lower altitudes had a higher likelihood of HPAI infection consistent with previous studies of factors influencing HPAI outbreaks in Thailand and China [4,10,12]. Other factors described as influencing HPAI outbreaks in other studies [12] and references therein, such as the proximity of farms to roads (ROADS), bodies of water (RIVER), feed markets (FEED), and slaughterhouses (SLAUGHTER), were found to be not significant using the spatial random effects logistic model.

**Figure 1:**
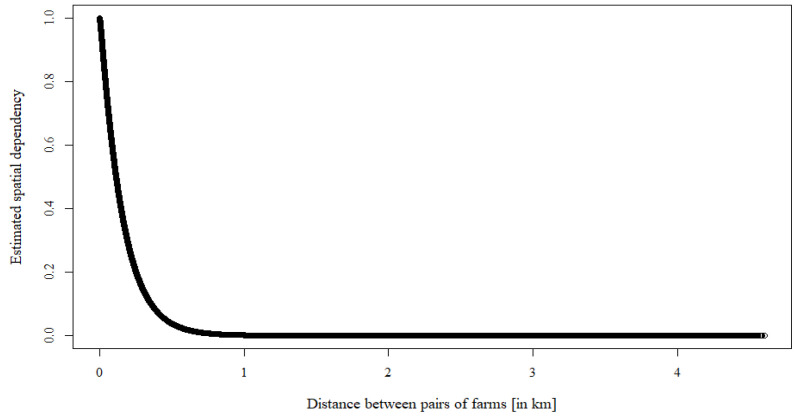
Spatially correlated random effects. As estimated from Equation (4) with respect to the distance between pairs of farms (km).

For the spatially correlated random effects, the coefficients of the smoothness parameter, ν, and the scale (correlation decay) parameter, ρ, are 0.5654 and 6.8985, respectively, which represent the strength and the speed of decay of the spatial random effect. The spatial correlation of the random effects dramatically decreases as the distance between pairs of poultry farms increases and approaches zero at approximately 0.75 km distance between farms (Figure 1).

### 3.2. Model Assessment

**Figure 2:**
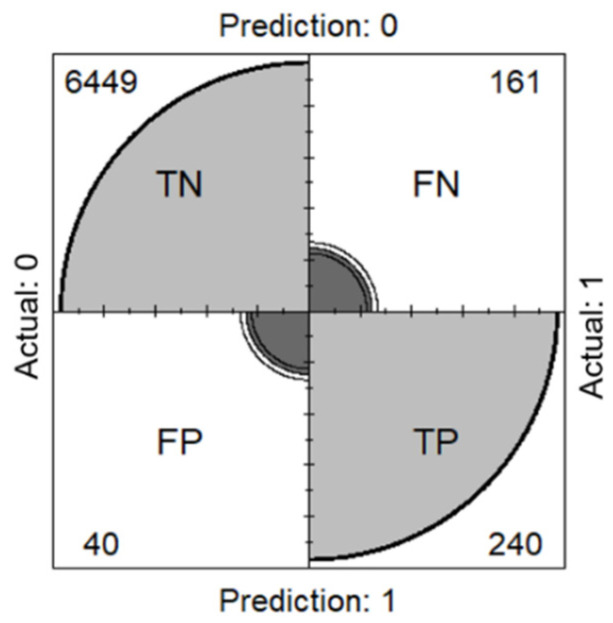
Confusion matrix for the farms being infected with HPAI. *TP* is a true positive, *TN* is a true negative, *FN* is a false negative, and *FP* is a false positive.

**Figure 3:**
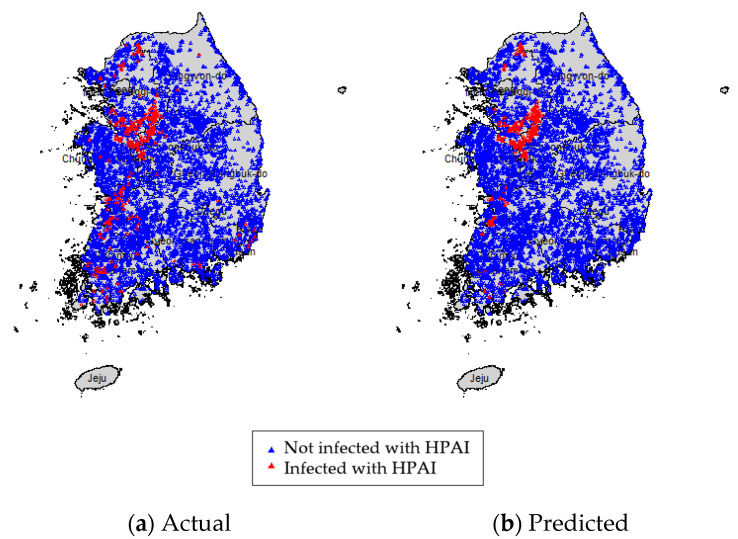
Spatial distribution of poultry farms. Actual (**a**) vs. Predicted (**b**) using the spatial random effects logistic model. Farms infected by HPAI are shown in red. Farms not infected by HPAI are shown in blue.

The predictive accuracy of the spatial random effects logistic model was 97%. Of the 6890 farms analyzed, 6689 farms (240 HPAI-infected farms and 6449 farms not infected with HPAI) were correctly identified (Figure 2). Of the 201 farms misclassified, 161 farms were false negatives, where a farm was predicted to be uninfected but was actually infected with HPAI, and 40 farms were false positives, where a farm was predicted to be infected but was not actually infected. Figure 3 illustrates a map of the poultry farms in Korea showing the actual (Figure 3a) versus predicted (Figure 3b) results from the spatial random effects logistic model for the 2016–2017 HPAI outbreak. Figure 3a,b are comparable, demonstrating that the model predicts the probability of a particular farm being infected with HPAI well.

### 3.3. Effective Culling Radius

HPAI is more of a concern for farms located closer to the initial HPAI-infected farm. To determine the effective culling radius, the spatial autocorrelation of the probability of a farm being infected with HPAI among neighboring farms was tested, and the Moran’s *I* statistics are presented in Table 4.

**Figure 4:**
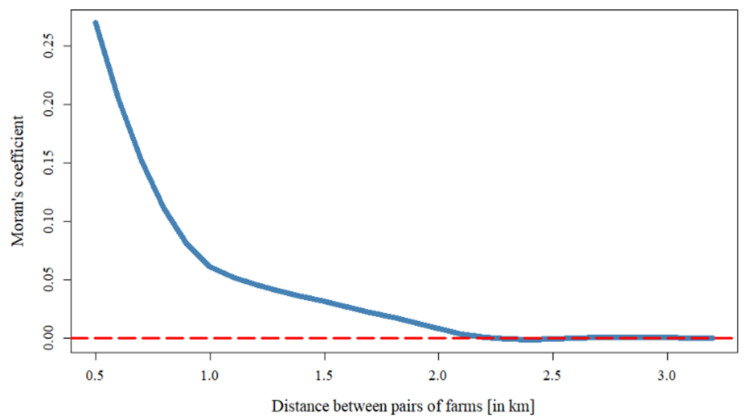
Moran’s *I* statistics for spatial autocorrelation of the predicted probability of a farm being infected with HPAI among neighboring farms with respect to the distance between pairs of farms (km).

The Moran’s *I* statistics gradually decrease as the distance between pairs of farms increases and converges to around zero at 2.2 km (Figure 4). The probability of a farm being infected from neighboring HPAI-infected farms is positively correlated from within 0.5 km to 2.2 km radii at the 1% significance level. From 2.3 km to 2.6 km radii, there was no significant spatial autocorrelation. The second range of radii was positively significant from 2.7 km to 3.0 km. At the 3.1 km radius, there was a significant negative correlation indicating spatial dispersion of the probability. Thus, the probability of a farm being infected with HPAI from neighboring infected farms was clustered within two ranges: from 0.5–2.2 km and 2.7–3.0 km.

## 4. Discussion

The occurrence of HPAI is caused by complex interactions of various factors, including the movement of people and vehicles, migratory birds, and breeding environments of the farms. The logistic regression model found factors, previously described as significantly associated with HPAI infection, to be significant. However, once the spatial random effects were controlled, a number of these factors were no longer significant. Specifically, factors such as the density of farms, density of livestock, and proximity of farms to roads, feed markets, or slaughterhouses were not significant using the spatial random effects logistic model. In this study, it was found that the poultry number of a farm and the number of poultry of the same type of breed within a county were associated with a higher probability of HPAI infection. The same type of poultry breed is consistent with what was found in Chungbuk among duck farms [21,23] as a contributing risk factor.

Susceptibility to HPAI infection previously attributed to physical distance may actually be capturing the frequency of agricultural activity resulting in HPAI spread by transport of infected materials, people, and/or vehicles. During the 2016–2017 HPAI outbreak, the countermeasures implemented to prevent the spread of HPAI included the quarantine of farms, vehicle checkpoints, decontamination protocols, and limiting the number of visits to farms by people and vehicles within the declared Emergency Quarantine Zones. Thus, the factors found not to be significant in this study such as proximity to roads, feed markets, slaughterhouses, and density of farms and poultry, may be due to the actions taken to prevent the further spread of HPAI during this outbreak.

This may explain the seemingly “contradictory” result found between the density of farms located within a 500 m radius (FARMS500m) and farms located in “mass breeding” sites defined as ≥10 farms in a 500 m radius or ≥20 farms in a 1 km radius. Farms located in “mass breeding sites” were significantly at greater risk of HPAI infection, not because of the greater density of farms, but perhaps because of the greater frequency of agricultural activity, a history of previous infections, or the increased monitoring present within these regions. Farms located in government-designated HPAI prevention districts (Intensive Quarantine Control Zones) include farms that are in “mass breeding sites” (MONITORED), and farms in proximity to habitats of migratory birds associated with HPAI infections. These farms are monitored because of their higher risk of HPAI infection. This study found that both of these farms have a greater probability of HPAI infection. Therefore, the designation of these Intensive Quarantine Control Zones and the increased precautionary measures and surveillance play a crucial role in detecting and preventing the spread of HPAI. These results suggest that current measures of monitoring and surveillance of these farms, implemented by the Korean government to detect and prevent HPAI outbreaks, are appropriate.

Two recent studies have estimated the effective culling radius based upon the 2016–2017 HPAI outbreak in Korea [21,23]. In both Yoo et al. [21] and Lee et al. [23], the analyses were focused upon the same general time period of the 2016–2017 outbreak and were constrained to Chungbuk in Korea containing higher densities of duck farms. Thus, conclusions from these previous studies may be particular to that region and time period during the 2016–2017 HPAI outbreak. Despite the differences in the scope of data analyzed and the methodologies employed among our study and the two previous studies, the estimation of the most effective culling radius is strikingly similar. We found two ranges within which spatial autocorrelation exists for the probability of a farm being infected with HPAI from neighboring farms. The first was from 0.5 km out to 2.2 km, and the second occurred from 2.7–3.0 km. Lee et al. [23] estimated an effective culling radius of 2.24 km, when not considering spatial heterogeneity, and 2.65 km when spatial heterogeneity, represented by the local reproductive numbers of the virus, was considered. Yoo et al. [21] simulated the risk for the local transmission of HPAI based upon the heterogeneity of the different clusters as determined by the genotype of the viruses and, although not conclusive, suggested that culling radii between 2–3 km are more appropriate, but only in areas with high densities of duck farms.

Limitations of this study include that the movement of people and vehicles in regions affected with HPAI were not considered, which may allow for the spread of HPAI over longer distances. Additionally, the temporal aspects of the outbreak and the epidemiological characteristics of the HPAI virus, such as transmissibility, reproduction number, and phylogenetic relatedness were not considered. Clusters of farms in close proximity may be affected by different introductions of HPAI at the same time, or infections from the same virus at different times. Additionally, the high standard deviations observed for some of the independent variables indicate the data as having a skewed distribution. This may have an effect on the robustness of the estimates. Future studies will address the spread of HPAI as influenced by these factors as well as other factors such as the level of farmers’ compliance with the quarantine control measures, the farming environment, implementation of on-farm biosecurity control measures, and movement of livestock vehicles.

## 5. Conclusions

Outbreaks of HPAI cause extremely large financial losses to the poultry industry and are a threat to human health. Determining the most effective control measures, especially the culling radius, to minimize economic impacts yet contain the spread of HPAI is of great importance. This study examines the factors influencing the probability of a farm being infected with HPAI during the 2016–2017 HPAI outbreak in Korea, and once estimated, determines the effective culling radius.

Once the spatial random effects are controlled for, many factors typically associated with increased risk of HPAI infection were no longer significant, although this may also reflect the effectiveness of the control measures implemented. Interestingly, farm density as measured within a radius of 500 m, 3 km, and 10 km from a farm, was not directly associated with increased risk of infection, but farms located in pre-determined “mass breeding sites”, defined as higher densities of farms, were significant.

The culling of poultry from HPAI-infected farms and surrounding farms is implemented to prevent the spread of HPAI, but the measures used in the 2016–2017 outbreak inflicted severe economic hardship upon the poultry industry and the Korean government. Based upon the spatial dependency of the predicted probability of HPAI infection, we found two ranges within which spatial autocorrelation exists for the probability of a farm being infected with HPAI from neighboring farms. The first was from 0.5 km out to 2.2 km, and the second occurred from 2.7–3.0 km. Thus, the effective culling radii were found to be 2.2 km and 3 km. These two ranges are consistent with previous studies examining the most effective culling radius of the 2016–2017 HPAI outbreak, and may reflect spatial heterogeneity, such as differences in transmissibility, local reproduction numbers, or different introductions of HPAI viruses [21,23]. Therefore, when considering what the culling radius should be, regional characteristics and the different types of HPAI virus(es) involved in the outbreak should be considered.

This study is valuable in that it analyzed the factors contributing to the risk of HPAI infection by constructing a spatial econometrics model that controls for the spatial random effects of HPAI occurrence. Additionally, it confirmed the 3 km culling radius for the 2016–2017 HPAI outbreak but found a smaller radius, 2.2 km, that may indicate spatial heterogeneity not considered in this study. The findings of this study will enable authorities to quickly respond to HPAI outbreaks with established quarantine countermeasures to suppress the spread of HPAI and help strengthen biosecurity control measures at the farm level.

## Figures and Tables

**Table 1:** Farms analyzed from the 2016–2017 HPAI outbreak.

Status of Farms		Total	Laying Hens	Broiler Hens	Breeder Hens	Broiler Ducks	Breeder Ducks
Infected	Yes	401	164	77	0	125	35
with HPAI	No	6489	1073	1934	348	3029	105
	Total	6890	1237	2011	348	3154	140
Monitored	Yes	168	108	13	5	38	4
farms	No	6722	1129	998	343	3116	136

**Table 2:** Descriptive statistics (*N* = 6890).

Variable	Description	Mean	Std. Dev.
Dependent variable	Dummy = 1 if a farm is infected with HPAI	0.058	0.234
HEADS	Number of poultry heads at each farm (1000 s)	21.659	49.543
BREED HEADS	Number of same breed heads in each county (1000 s)	467.520	770.751
COUNTY HEADS	Number of poultry heads in each county (1000 s)	1374.393	1121.574
HEADS500m	Number of poultry heads within 500 m from each farm (1000 s)	36.727	87.333
HEADS3km	Number of poultry heads within 3 km from each farm (1000 s)	135.782	209.968
HEADS10km	Number of poultry heads within 10 km from each farm (1000 s)	790.685	782.061
FARMS500m	Number of poultry farms within 500 m from each farm	1.607	1.770
FARMS3km	Number of poultry farms within 3 km from each farm	5.726	4.456
FARMS10km	Number of poultry farms within 10 km from each farm	34.090	18.145
MONITERED	Dummy = 1 if a farm is in a mass breeding site	0.024	0.154
ROAD	Distance to road (km)	0.602	0.684
FEED	Distance to feed market (km)	4.571	3.704
SLAUGHTER	Distance to slaughterhouse (km)	24.231	20.287
RIVER	Distance to rivers or lakes (km)	0.789	0.875
MIGRATORY	Distance to habitats of migratory birds (km)	29.891	18.898
ALTITUDE	Altitude (m)	119.046	122.562

**Table 3:** Estimation results and probability of a farm being infected with HPAI.

	Logistic Regression Model		Spatial Random Effects Logistic Model	
Variable	Coefficient	Std. Error	Coefficient	Std. Error
Constant	−1.2650 ***	(0.2824)	−3.6140 ***	(0.7421)
HEADS	0.0042 ***	(0.0015)	0.0070 ***	(0.0022)
BREED HEADS	0.0003 ***	(0.0001)	0.0003 ***	(0.0001)
COUNTY HEADS	0.0000	(0.0001)	−0.0001	(0.0001)
HEADS500m	−0.0010	(0.0012)	−0.0029	(0.0018)
HEADS3km	0.0010	(0.0004)	0.0010 *	(0.0006)
HEADS10km	0.0009 ***	(0.0001)	0.0003	(0.0003)
FARMS500m	−0.0910	(0.0626)	0.0197	(0.1026)
FARMS3km	0.0693 ***	(0.0205)	0.0032	(0.0344)
FARMS10km	−0.0213 ***	(0.0058)	0.0091	(0.0135)
MONITERED	1.8270 ***	(0.2756)	1.3600 **	(0.5447)
ROAD	−0.3390 ***	(0.1311)	0.0087	(0.1739)
FEED	−0.0895 ***	(0.0288)	0.0022	(0.0467)
SLAUGHTER	−0.0454 ***	(0.0071)	−0.0094	(0.0152)
RIVER	−0.0070	(0.0800)	0.0524	(0.1166)
MIGRATORY	−0.0585 ***	(0.0057)	−0.0391 **	(0.0166)
ALTITUDE	0.0010	(0.0009)	−0.0056 **	(0.0025)
ν			0.5654	—
ρ			6.8985	—

Notes: *, **, *** represent statistical significance at the 10%, 5%, and 1% levels. Standard errors are shown in parenthesis.

**Table 4:** Moran’s *I* statistics. Spatial autocorrelation of the predicted probability of a farm being infected with HPAI among neighboring farms with respect to the distance between pairs of farms (km).

Distance (km)	Moran’s *I*	*p*-Value	Distance (km)	Moran’s *I*	*p*-Value
0.5	0.2699 ***	0.0000	1.9	0.0128 ***	0.0000
0.6	0.2043 ***	0.0000	2.0	0.0079 ***	0.0000
0.7	0.1514 ***	0.0000	2.1	0.0035 ***	0.0000
0.8	0.1110 ***	0.0000	2.2	0.0007 ***	0.0000
0.9	0.0797 ***	0.0000	2.3	−0.0008	1.0000
1.0	0.0608 ***	0.0000	2.4	−0.0015	1.0000
1.1	0.0517 ***	0.0000	2.5	−0.0011	1.0000
1.2	0.0460 ***	0.0000	2.6	−0.0001	0.4899
1.3	0.0399 ***	0.0000	2.7	0.0003 ***	0.0000
1.4	0.0355 ***	0.0000	2.8	0.0005 ***	0.0000
1.5	0.0312 ***	0.0000	2.9	0.0005 ***	0.0000
1.6	0.0268 ***	0.0000	3.0	0.0003 ***	0.0000
1.7	0.0218 ***	0.0000	3.1	−0.0000 ***	0.0007
1.8	0.0175 ***	0.0000	3.2	−0.0003	0.9998

Notes: *** represent statistical significance at the 1% levels.

## Data Availability

Data are available upon reasonable request from the corresponding author.
